# Urinary Deposits; Their Diagnosis, Pathology, and Therapeutical Indications

**Published:** 1851-08

**Authors:** 


Art. IX.—*Urinary Deposits;* their Diagnosis, Pathology, and Therapeutical Indications. By Golding Bird, A-M., M.D., F.R.S., F.L.S., etc., etc. Second American, from the third revised and enlarged London edition. Philadelphia: Blanchard & Lea. 1851.

This able and thorough work on urinary deposits should be well studied by all who attempt to teach or practice medicine. Its developments are of the greatest importance in the treatment of a large variety of diseases, and the more intimate the acquaintance, possessed by the physician, of the morbid changes of the organs concerned in the urinary function, the greater will be his power over the diseases dependent on these changes. There are few physicians who have much to do with disease, who will not find the knowledge we have indicated a matter of daily concern, and of the highest importance. The experiments of M. Bernard, of which we recently published an account, show how useful we may make therapeutic means, by a proper understanding of them, but there can be no secure basis for therapeutic agents, that is not founded upon an accurate acquaintance with the pathological conditions of the urine. In order to obtain an introduction to the investigations that will conduct to this knowledge, and to this usefulness, we commend to the physician a diligent study of Dr. Golding Bird's work on urinary deposits, and then with chemical manipulations and microscopic observations, he may prepare himself for relieving many diseases that are altogether obscure, without the means of analysis we have indicated.

In the preface to the third edition Dr. Bird says: "Anxious not to increase its bulk, I have rewritten rather than added; and in offering this edition to the notice of my profe-sional brethren, it is my earnest desire that it may be found more worthy of the favor and patronage they have so generally accorded to the two previous impressions."

A large number of wood engravings illustrate the various forms of morbid products of the urine, and they will be found of great utility to those who may engage in microscopic examinations.

These various works may be found at the bookstore of Messrs. Beckwith & Morton. B.

